# American Dental Association

**Published:** 1882-07

**Authors:** 


### AMERICAN DENTAL ASSOCIATION.

The twenty-second annual session will be held in Cincinnati, August 1st to 4th inclusive.

The meeting will be held in the Hall of the Highland House, situated on Mt. Adams. This Hall has been selected because it is a fine, capacious one, with side rooms for committees, etc. It is about five hundred feet above the city, is always cool and pleasant, even in the warmest weather. It is easily accessible, being within a few minutes ride by street and inclined railway.

Arrangements have been made for first-class hotel accommodations at $2.50 per day. Dinner can be taken at the Highland House by all who wish it. The Gibson House, on Walnut St,, between Fourth and Fifth Sts., will be headquarters for those attending the Association. Street cars pass the door every two or three minutes, going direct to the Highland House.

### TRANSPORTATION.

A reduction of rates has been secured on the following railroads: The Ohio & Miss. R.R. will return those attending the meeting to St. Louis, for $3.90, to Louisville for $1.50, and to all intermediate points at the rate of one cent per mile, on certificate of Secretary, that they paid full fare coming to Cincinnati.

The Cincinnati & Louisville Short Line R.R. agree to the same rates between Louisville and Cincinnati.

The New York, Penn. & Ohio R'y, from Salamanca to Cincinnati, will return visitors at one cent per mile, on certificate of the Secretary.

Cincinnati, Indianapolis, St. Louis & Chicago R'y, "Kankakee Line," between Cincinnati and Chicago, "Big Four and Vandalia Line" between Cincinnati and St. Louis, will return those attending the meeting at—$3.00 to Chicago, $3.90 to St. Louis, and one cent per mile to Indianapolis, Lafayette, Kankakee, and intermediate stations, on certificate that they paid full fare going.

The Cincinnati,. Hamilton & Dayton R.R. will make same rates as other routes to Indianapolis, Richmond, Chicago, Toledo, etc.

The C. C. C. & I. R.R., from Cleveland to Cincinnati, will also return all attending the Association at one cent per mile.

The Cincinnati Southern R.R. will return those who have paid full fare coming, for two cents per mile.

The Pittsburgh, Cincinnati & St. Louis R.R. (Pan Handle Route), will furnish round-trip tickets, at two cents per mile; from Pittsburgh to Cincinnati and all intermediate points to all who may present orders for the same. These orders for round-trip tickets may be obtained by addressing J. Taft, Cincinnati, O.

The Baltimore & Ohio R.R. will sell excursion tickets between Parkersburg and Cincinnati, and all intermediate points, at two cents per mile, each way, to those attending the Association.

The Chesapeake & Ohio R.R., extending to Washington, D. C., and Richmond, Va., reaching Cincinnati, *via* Lexington, Ky., on the Kentucky Central R.R., will return those attending the Association from these and all intermediate points reached by this railroad and its branches, at one cent per mile, upon certificate of the Secretary of the Association. These tickets will be good for stopping over at any point of resort or interest on the road. The White Sulphur Springs are on this route

J. TAFT,
G. W. KEELY, } Com. of Arrangements.
C. R. BUTLER,

